# Large-scale fabrication of porous YBO_3_ hollow microspheres with tunable photoluminescence

**DOI:** 10.1098/rsos.172186

**Published:** 2018-04-11

**Authors:** Zhenhe Xu, He Yu, Feixue Ai, Guiyan Zhao, Yanfeng Bi, Liangliang Huang, Fu Ding, Yaguang Sun, Yu Gao

**Affiliations:** 1 College of Chemistry, Chemical Engineering and Environmental Engineering, Liaoning Shihua University, Fushun 113001, People's Republic of China; 2 The Key Laboratory of Inorganic Molecule-Based Chemistry of Liaoning Province, College of Applied Chemistry, Shenyang University of Chemical Technology, Shenyang 110142, People's Republic of China

**Keywords:** luminescence, rare earth compounds, hollow microspheres, yttrium orthoborate

## Abstract

Hollow lanthanide-doped compounds are some of the most popular materials for high-performance luminescent devices. However, it is challenging to find an approach that can fabricate large-scale and well-crystallized lanthanide-doped hollow structures and that is facile, efficient and of low cost. In this study, YBO_3_: Eu^3+^/Tb^3+^ hollow microspheres were fabricated by using a novel multi-step transformation synthetic route for the first time with polystyrene spheres as the template, followed by the combination of a facile homogeneous precipitation method, an ion-exchange process and a calcination process. The results show that the as-obtained YBO_3_: Eu^3+^/Tb^3+^ hollow spheres have a uniform morphology with an average diameter of 1.65 µm and shell thickness of about 160 nm. When used as luminescent materials, the emission colours of YBO_3_: Eu^3+^/Tb^3+^ samples can be tuned from red, through orange, yellow and green-yellow, to green by simply adjusting the relative doping concentrations of the activator ions under the excitation of ultraviolet light, which might have potential applications in fields such as light display systems and optoelectronic devices.

## 1. Introduction

Luminescent materials, especially lanthanide-doped materials, have attracted extensive synthetic interest due to their remarkable luminescence properties, such as various emission colours, high photochemical stability, low toxicity, narrow emission peaks and large anti-Stokes shifts [1–8]. These advantages lead to their excellent performance in versatile applications such as lighting, information display technologies, solar cells, biological labelling and biomedical imaging technology [9–13]. Among lanthanide-doped materials, yttrium orthoborate (YBO_3_) is one category of useful host lattices for luminescence [14]. Thanks to the high damage threshold, high-vacuum ultraviolet (VUV) transparency and nonlinear optical efficiency resulting from the B–O structure, it exhibits extraordinarily high luminescence efficiency under VUV excitation and it is considered to be attractive candidate as VUV luminescent material, which has found applications in Hg-free fluorescent lamps and plasma display panels [15–18]. Up to now, YBO_3_ micro/nanocrystals with abundant morphologies have been synthesized through a variety of techniques, such as hydro/solvothermal techniques [19], vapour–liquid–solid method, electrospinning method [20], sol–gel routes and solid-phase method. Through these techniques, various morphologies of YBO_3_ materials have been synthesized, such as well-dispersed nanocrystals [21], one-dimensional nanowires and nanotubes [20], drum-like microcrystals [22] and three-dimensional flower-like architectures [23].

As a unique family of functional materials, hollow structure materials possess a large fraction of empty space and high surface area which endow them with broad applications in gas sensors, drug delivery, biomaterials, water treatment, supercapacitors, dye-sensitized solar cells, heterogeneous catalysts, fuel cells, etc. [4,9,24–31]. Thus, much attention has been devoted to synthesizing hollow structures of various functional materials. Basically, there are four main methods for synthesizing hollow structures: (i) conventional hard templating method, (ii) sacrificial templating method, (iii) soft templating method and (iv) template-free method. Among all these commonly used strategies, hard templating method is an established, industrially relevant, simple and scalable protocol to produce hollow materials [32]. Basically, the preparation of hollow structures using hard templating method consists typically of three steps: (i) template preparation, (ii) coating of the template and (iii) removal of the template [33–35]. Although there are a lot of reports on hard templating synthesis of hollow structures, including C_3_N_4_ [36], Ta_3_N_5_ [37], TiO_2_ [38], ZnO [39], BiMoO_6_ [40] and LaTiO_2_N [41], reports on YBO_3_ hollow structure are very rare. Furthermore, as is well known, YBO_3_ has been proved to be a very efficient host lattice for the luminescence of Eu^3+^ and Tb^3+^ ions. However, based on our knowledge, up to now, there is no report available on the preparation of Eu^3+^- and Tb^3+^-co-doped YBO_3_ hollow structure which shows tunable luminescence properties. Generally speaking, developing facile, cost-effective, environmentally friendly and scalable strategies for the synthesis of YBO_3_ spherical hollow structure is still a key challenge.

Herein, we used polystyrene (PS) microspheres as the template to synthesize YBO_3_ hollow spheres via the combination of a homogeneous precipitation method, an ion-exchange process and a calcination process. Besides, we also systematically investigated the photoluminescence (PL) colours of the YBO_3_ hollow spheres co-doped with Eu^3+^ and Tb^3+^ ions, which could be tuned from red, through yellow and green-yellow, to green by simply adjusting the relative doping concentrations of the activator ions. The development of this method would offer a new platform for the fabrication of other hollow structure materials.

## 2. Experimental

### 2.1. Materials

The rare earth oxides Y_2_O_3_ (99.99%), Eu_2_O_3_ (99.99%), Tb_4_O_7_ (99.99%) and other chemicals were purchased from Aladdin Reagent Co. Ltd. Rare earth chloride stock solutions were prepared by dissolving the corresponding metal oxide in hydrochloric acid at an elevated temperature. All chemicals were analytical-grade reagents and used as purchased without further purification.

### 2.2. Preparation of monodispersed polystyrene microspheres

In a typical synthesis, the poly(*N*-vinylpyrrolidone) K30 stabilizer (1.0 g) was dissolved in ethanol (38.2 ml) in a three-necked round bottom flask fitted with a condenser and a magnetic stirrer. The reaction vessel was then heated to 70°C under a nitrogen blanket and purged with nitrogen for 2 h. Then, a solution of azoisobutyronitrile (0.15 g) pre-dissolved in styrene monomer (15 g) was added to the reaction vessel with vigorous stirring. The styrene polymerization was allowed to proceed for 12 h before cooling to room temperature. The product was purified by repeated centrifugation and washed with ethanol. A white fine powder (PS) was finally obtained after being dried in a vacuum oven at 50°C.

### 2.3. Preparation of the monodisperse YBO_3_ hollow microspheres

First, 1 mmol of YCl_3_ (0.2 M) aqueous solution and the as-prepared PS microspheres (100 mg) were added to 50 ml deionized water and well dispersed with the assistance of ultrasonication for 30 min. Then, 2.0 g of urea was dissolved in the solution under vigorous stirring. Finally, the mixture was transferred into a 100 ml flask and heated at 90°C for 2 h with vigorous stirring before the product was collected by centrifugation. The product was washed with deionized water and ethanol three times. Second, the as-obtained sample was dispersed in deionized water by ultrasonication for 30 min. Then, 0.2 g of H_3_BO_3_ dissolved in an appropriate amount of deionized water was dripped into the dispersion followed by further stirring. After additional agitation for 60 min, the as-obtained mixing solution was transferred into a Teflon bottle held in a stainless steel autoclave, sealed and maintained at 180°C for 24 h. As the autoclave was cooled to room temperature naturally, the precipitate was separated by centrifugation, washed with deionized water and ethanol in sequence, and then dried in air at 80°C for 12 h. Finally, the final YBO_3_ hollow microspheres were obtained through a heat treatment at 800°C in air for 4 h with a heating rate of 1°C min^−1^. The YBO_3_: Eu^3+^/Tb^3+^ hollow microspheres were prepared in a similar procedure except that by adding corresponding EuCl_3_ and TbCl_3_ together with YCl_3_ as the starting materials as described above.

### 2.4. Characterization

Powder X-ray diffraction (XRD) measurement was performed with a Rigaku-Dmax 2500 diffractometer with Cu K*α* radiation (*λ* = 0.15405 nm). Raman spectra were obtained by a Lab RAM HR system of Horiba JobinYvon at room temperature using a 532 nm solid-state laser as excitation source. Thermogravimetric analysis (TGA) data were recorded with a thermal analysis instrument (SDT 2960, TA Instruments, New Castle, DE, USA) with a heating rate of 10°C min^−1^ in an air flow of 100 ml min^−1^. The morphologies and composition of the as-prepared samples were inspected with a field emission scanning electron microscope (SEM, SU8010, Hitachi). Low- to high-resolution transmission electron microscopy (TEM) was performed using an FEI Tecnai G^2^ S-Twin with a field emission gun operating at 200 kV. Images were acquired digitally with a Gatan multiople CCD camera. The PL excitation and emission spectra were recorded with a Hitachi F-4500 spectrophotometer equipped with a 150 W xenon lamp as the excitation source. All measurements were performed at room temperature.

## 3. Results and discussion

**Figure 1.**
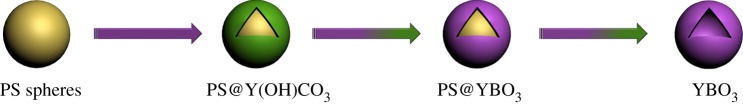
Schematic of the synthesis route of the YBO_3_ hollow microspheres.

The strategy of preparing the YBO_3_ hollow microspheres is highly repeatable and revealed in figure 1. The whole process can be mainly divided into four steps. (i) Synthesis of the well-monodisperse PS colloidal microspheres by dispersion polymerization [42]. (ii) Synthesis of the core-shell PS@Y(OH)CO_3_ microspheres by the homogeneous precipitation method using urea as the precipitating agent. The PS microspheres have a lot of hydroxyl groups, which are beneficial to the adsorption of Y^3+^, OH^−^ and ${\text{CO}}_{3}^{2-}$ (released from precipitator agent urea) (equations (3.1)–(3.3)). (iii) Formation of the core-shell PS@YBO_3_ microspheres by an ion-exchange process under hydrothermal condition. Under hydrothermal process, the H_3_BO_3_ is able to react with Y(OH)CO_3_ to form some YBO_3_ nanoparticles (equation (3.4)). Subsequently, the interface chemical transformation gradually continued to occur with the inner layer in the hydrothermal condition, resulting in the pure YBO_3_ layer. (iv) Calcination of the core-shell PS@YBO_3_ microspheres in air to remove the PS microsphere template to get the YBO_3_ hollow spheres and increase of crystallinity of the final product. The main chemical reactions for the formation of the YBO_3_ hollow microspheres could be represented as follows:

3.1 $$\begin{array}{r} \text{CO}{\left(\text{N}{\text{H}}_{2}\right)}_{2}+{\text{H}}_{2}\text{O}⇌\text{C}{\text{O}}_{2}+2\text{N}{\text{H}}_{3}, \end{array}$$

3.2 $$\begin{array}{r} \text{N}{\text{H}}_{3}+{\text{H}}_{2}\text{O}⇌\text{N}{{\text{H}}_{4}}^{+}+\text{O}{\text{H}}^{-}, \end{array}$$

3.3 $$\begin{array}{r} \text{PS}+{\text{Y}}^{3+}+\text{O}{\text{H}}^{-}+\text{C}{{\text{O}}_{3}}^{2-}⇌\text{PS}@\text{Y( OH) C}{\text{O}}_{3} \end{array}$$

3.4 $$\begin{array}{r} \;\text{and}\;\text{PS}@\text{Y}(\text{OH})\text{C}{\text{O}}_{3}+3{\text{H}}^{+}+\text{B}{{\text{O}}_{3}}^{3-}⇌\text{PS}@\text{YB}{\text{O}}_{3}+2{\text{H}}_{2}\text{O}+\text{C}{\text{O}}_{2}. \end{array}$$

**Figure 2.**
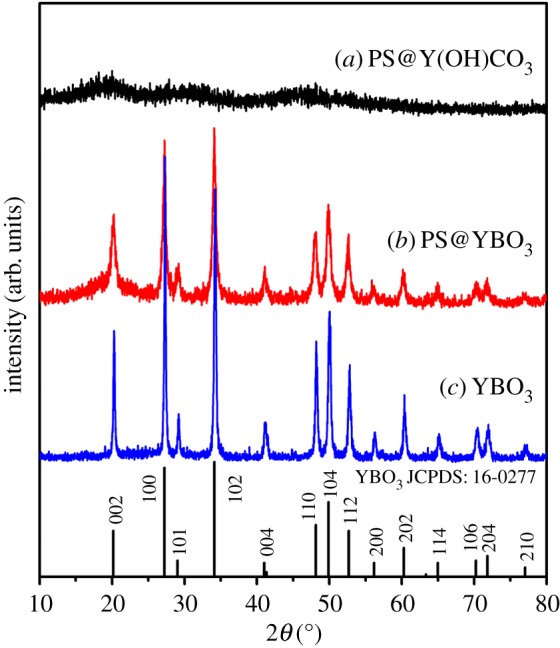
XRD patterns of (*a*) the core-shell PS@Y(OH)CO_3_ microspheres, (*b*) the core-shell PS@YBO_3_ microspheres and (*c*) the YBO_3_ hollow microspheres.

**Figure 3.**
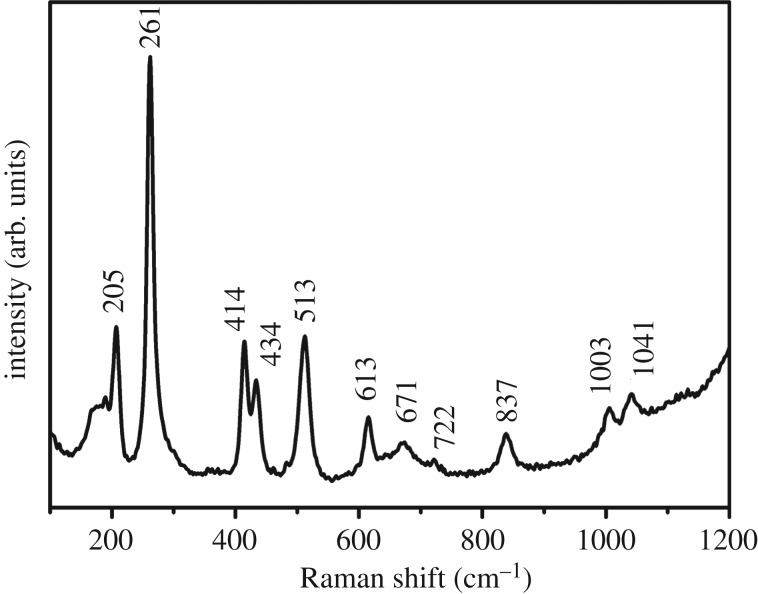
Raman spectrum of the YBO_3_ hollow microspheres at room temperature.

The phase purity and crystal structure of the obtained samples were examined by XRD (figure 2). After the homogeneous precipitation reaction, no obvious diffraction peak appears in the pattern of the sample (PS@Y(OH)CO_3_), indicating that the as-formed core-shell PS@Y(OH)CO_3_ sample is amorphous. After the hydrothermal reaction, the diffraction pattern of the sample can be indexed to the hexagonal-vaterite phase of YBO_3_ (JCPDS no. 16-0277, space group *P*6_3_*/m*, *z* = 2 and cell parameter *α* = 3.778 Å, *c* = 8.806 Å). After annealing at 800°C for 4 h, all of the diffraction peaks can also be well indexed to the tetragonal phase of YBO_3_, and no other impurity peaks can be detected, indicating the formation of a purely YBO_3_ phase. It can also be seen that the diffraction peaks of the YBO_3_ sample are very sharp and strong, revealing that the YBO_3_ product with high crystallinity can be synthesized using this method. This is important for phosphors because high crystallinity generally means fewer traps and stronger luminescence. In order to further confirm the structure, a typical Raman spectrum of YBO_3_ sample recorded at room temperature is shown in figure 3. The Raman peaks in the low wavenumber region, such as 185, 196 and 205 cm^−1^, should be related to the translations of the Y^3+^ cations and the B_3_O_9_ groups, and the vibrational modes of the B_3_O_9_ groups. The other bands in the 250–1200 cm^−1^ region are related to the internal modes of the B_3_O_9_ groups. Furthermore, some splits of the internal vibrational bands should be attributed to the crystal field effect which may reduce the site symmetry of the B_3_O_9_ groups [43].

**Figure 4.**
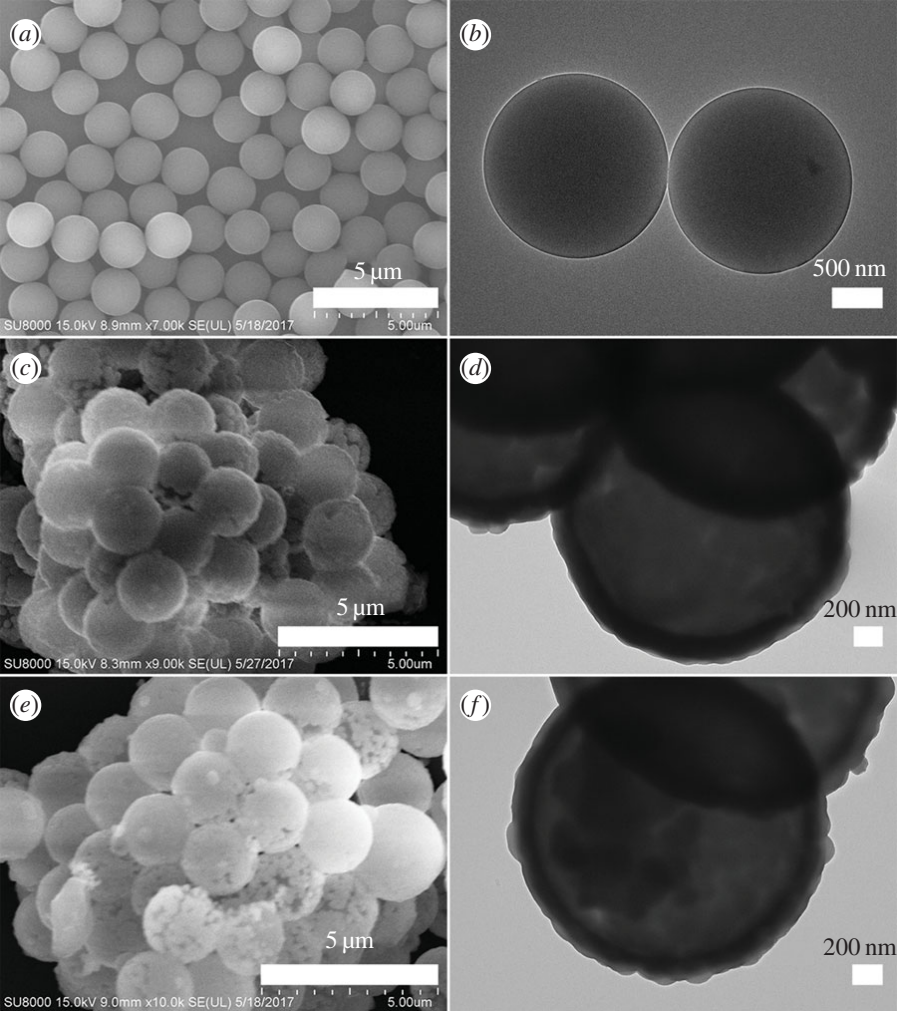
SEM and TEM images of (*a*,*b*) the PS spheres, (*c*,*d*) the core-shell PS@Y(OH)CO_3_ microspheres and (*e*,*f*) the core-shell PS@YBO_3_ microspheres.

The size and morphology of the products were further examined by SEM and TEM measurements. Figure 4*a*,*b* shows that the PS microspheres consist of well-dispersed microspheres with an average size of 1.85 µm and their surfaces are smooth. After the homogeneous precipitation reaction, the Y(OH)CO_3_ layers were coated around the PS microspheres (denoted as PS@Y(OH)CO_3_). From the SEM image (figure 4*c*), it can be seen that the sample inherits the spherical morphology, and the surfaces are much rougher than those of the PS microsphere template because of the precipitation of a large amount of nanoparticles. The size of the PS@Y(OH)CO_3_ is about 2.20 µm. Furthermore, detailed morphological identification was performed using TEM image analysis. Figure 4*d* presents a typical representative TEM image of the PS@Y(OH)CO_3_ sample, which consists of rough surface microspheres and the core-shell structures can be easily found via different colours of core and shell. The average size of the as-prepared sample is 2.20 µm in diameter and the thickness of the shell is about 175 nm. So the size of the PS@Y(OH)CO_3_ microspheres is larger than that of the pure PS microspheres, which further confirms the formation of the Y(OH)CO_3_ layer. When the PS@Y(OH)CO_3_ core-shell microspheres were treated with H_3_BO_3_ under hydrothermal conditions at 180^o^C for 24 h, the product (denoted as PS@YBO_3_) largely inherits the shape and dimension of the PS@Y(OH)CO_3_ core-shell microspheres (figure 4*e*). The size of the product is similar to the core-shell PS@Y(OH)CO_3_ microspheres in the size range of 2.20 µm. From the TEM image (figure 4*f*), it can be seen that the average size of the core-shell microspheres is about 2.20 µm and the shell thickness is about 175 nm, which conforms to the size calculated from the SEM image.

**Figure 5.**
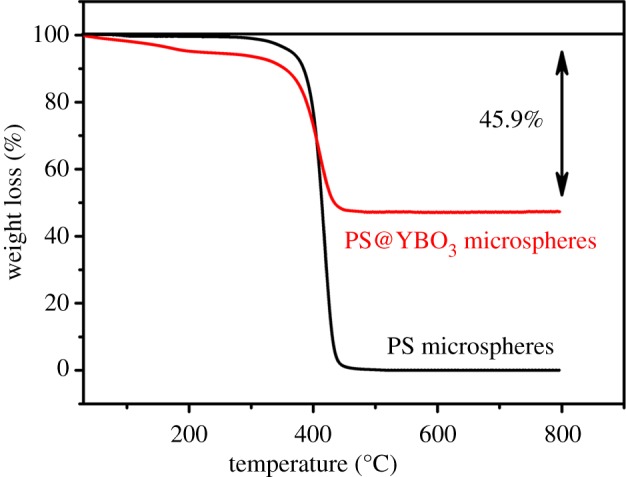
TGA curves of the PS spheres and the core-shell PS@YBO_3_ microspheres.

**Figure 6.**
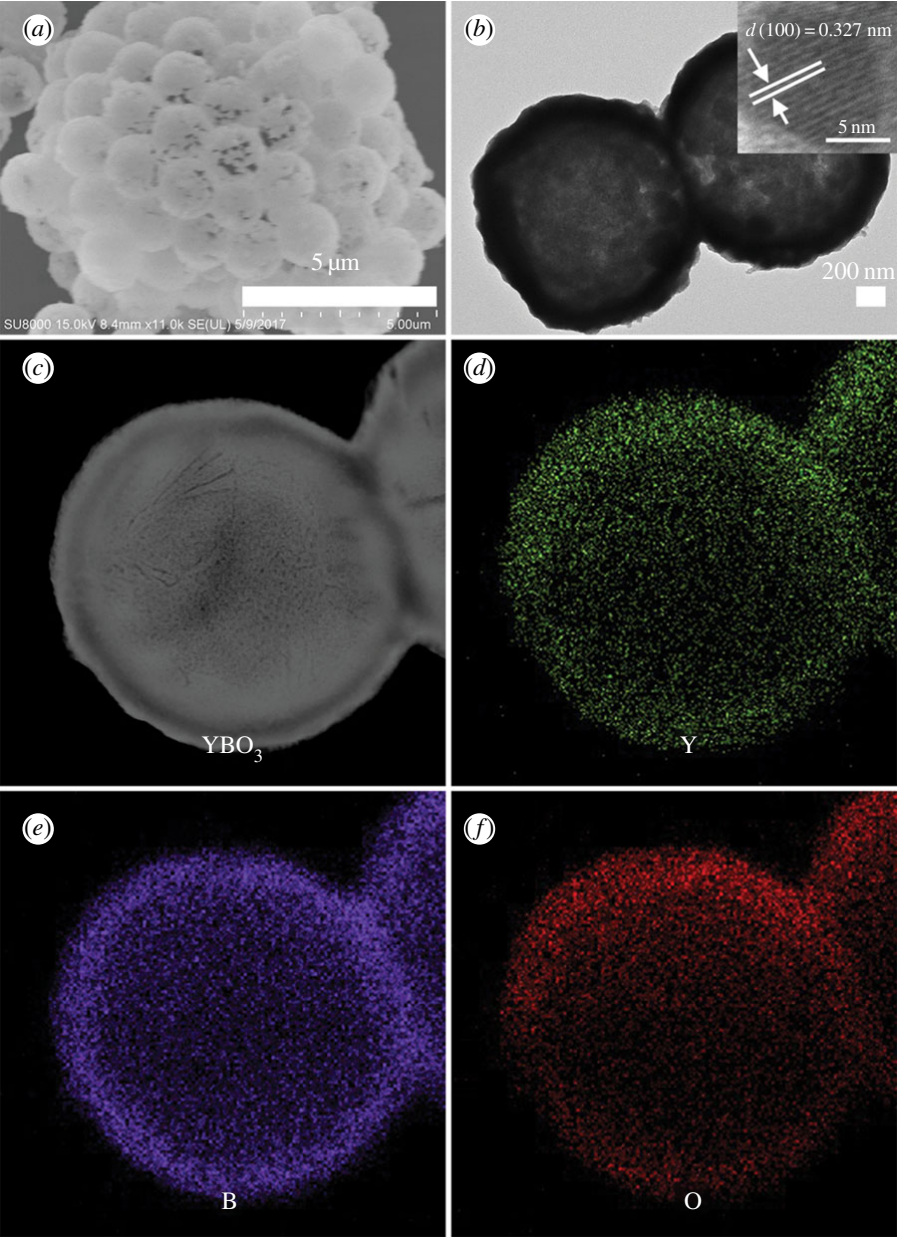
(*a*) SEM, (*b*) TEM and (*b*, inset) high-resolution TEM images of the YBO_3_ hollow microspheres. (*c*) HAADF-STEM image of the YBO_3_ hollow microspheres and the corresponding elemental maps for (*d*) Y, (*e*) B and (*f*) O.

Thermal decomposition of the PS microsphere template is a simple and conventional route to form a hollow structure. After the synthesis of the core-shell PS@YBO_3_ microspheres, we also investigated the effect of calcination on the morphology of the as-prepared product. Figure 5 shows the TGA curves of the PS microspheres and the core-shell PS@YBO_3_ microspheres. For the PS microspheres (black line), there is one weight loss which is attributed to the splitting burning of PS microspheres. For the core-shell PS@YBO_3_ microspheres, there are two stages of weight loss (red line): one is a slow weight loss because of the dehydration and densification of the PS microspheres. The other one is the burning of the PS microspheres. Finally, the residual weight percentage is about 54.1%, which accounts for the final YBO_3_ hollow microspheres, suggesting the considerably high yield of the hollow phosphors prepared using this method. So it can be concluded that the calcination process has a dual function: elimination of the PS microsphere cores to form hollow microspheres and the increase of crystallinity of the final product. The morphology, microstructure and elemental composition of YBO_3_ sample were revealed by SEM, TEM and high angle annular dark field scanning transmission electron microscopy (HAADF-STEM) (figure 6). The YBO_3_ sample exhibits sphere-like structure with a diameter of approximately 1.65 µm (figure 6*a*). In particular, the hollow microspheres can be clearly visualized from the rupture of one sphere with a typical wall thickness of around 160 nm (figure 6*a*). The sharp contrast between the edge and centre part of the hollow structure is clearly visible in the TEM image (figure 6*b*). The measured *d* spacing of 0.327 nm in the high-resolution TEM image (inset in figure 6*b*) can be indexed to the lattice spacing of the (100) plane of YBO_3_. In order to investigate the elemental distribution, HAADF-STEM image and elemental maps were acquired for an individual sphere (figure 6*c*–*f*). Elements Y, B and O are evenly distributed throughout the entire sphere, revealing that the YBO_3_ hollow sphere can be synthesized by the combination of a facile homogeneous precipitation method, an ion-exchange process and a calcination process.

**Figure 7.**
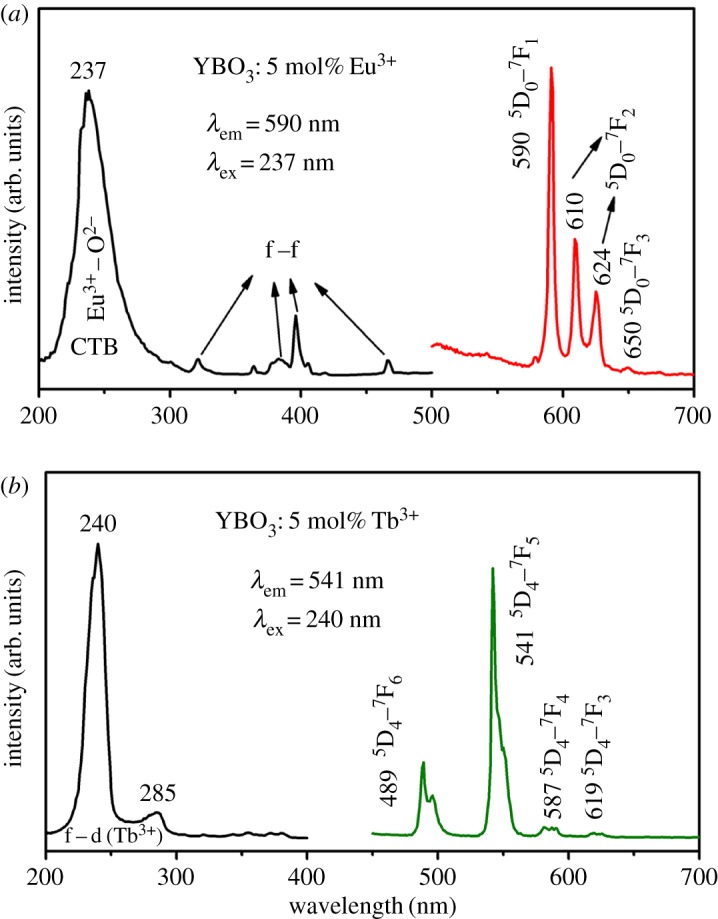
Photoluminescence excitation and emission spectra of as-prepared (*a*) YBO_3_: 5 mol% Eu^3+^ and (*b*) YBO_3_: 5 mol% Tb^3+^.

It is well known that Eu^3+^ or Tb^3+^ ions-doped YBO_3_ samples can emit strong red or green emission under UV excitation, respectively. The excitation and emission spectra of the YBO_3_: 5 mol% Eu^3+^ sample are shown in figure 7*a*. By means of monitoring at 590 nm, it was found that the excitation spectrum is composed of a strong absorption band centred at 237 nm and some weak lines, which are due to the charge transfer band between the O^2−^ and Eu^3+^ ions and f–f transition of the Eu^3+^ ions, respectively. Upon excitation at 237 nm, the emission spectrum of the YBO_3_: 5 mol% Eu^3+^ sample displays four palpable peaks, which are centred at 590, 610, 624 and 650 nm. The four lines correspond to the ^5^D_0_ → ^7^F_1_, ^5^D_0_ → ^7^F_2_, ^5^D_0_ → ^7^F_2_ and ^5^D_0_ → ^7^F_3_ transitions of Eu^3+^ ions in YBO_3_, respectively. The most prominent emission peak, attributed to the ^5^D_0_ → ^7^F_1_ transition of Eu^3+^, is located at 590 nm.

Figure 7*b* shows the excitation and emission spectra of the YBO_3_: 5 mol% Tb^3+^ sample. The excitation spectrum of the YBO_3_: 5 mol% Tb^3+^ sample monitored with 541 nm consists of two intense bands and some weak lines. The intense bands centred at 240 and 285 nm are attributed to the spin-allowed transition (Δ*S *= 0) with higher energy and the spin-forbidden transition (Δ*S *= 1) with lower energy from the 4f to the 5d level of the Tb^3+^ ions, respectively [16,44]. The other weak lines are due to the characteristic f–f transitions of the Tb^3+^ ions. The emission spectrum consists of a group of lines centred at about 489, 541, 587 and 619 nm, which correspond to the ^5^D_4_ → ^7^F*_J_* (*J *= 6, 5, 4, 3) transitions of the Tb^3+^ ions, respectively. The strongest one is located at 541 nm, corresponding to the ^5^D_4_ → ^7^F_5_ transition of Tb^3+^.

**Figure 8.**
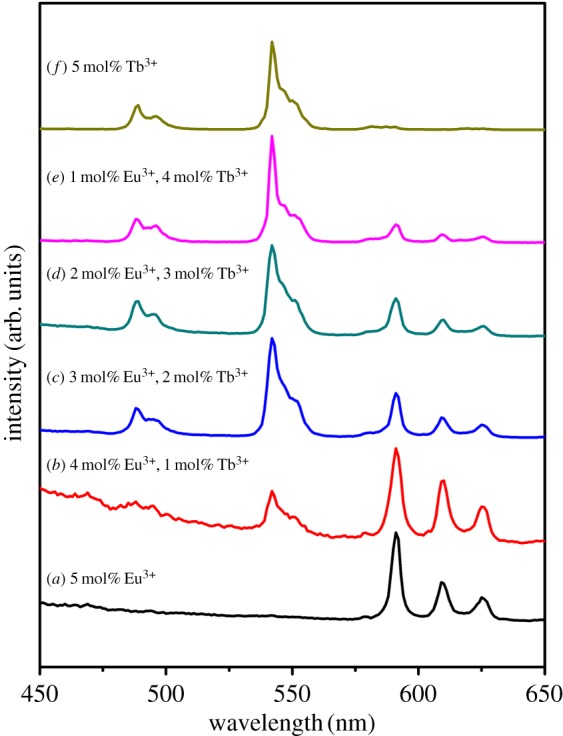
Photoluminescence emission spectra of the Eu^3+^ and Tb^3+^ co-doped YBO_3_ samples under excitation at 240 nm (total concentration: 5 mol%): (*a*) YBO_3_: 5 mol% Eu^3+^; (*b*) YBO_3_: 4 mol% Eu^3+^, 1 mol% Tb^3+^; (*c*) YBO_3_: 3 mol% Eu^3+^, 2 mol% Tb^3+^; (*d*) YBO_3_: 2 mol% Eu^3+^, 3 mol% Tb^3+^; (*e*) YBO_3_: 1 mol% Eu^3+^, 4 mol% Tb^3+^; (*f*) YBO_3_: 5 mol% Tb^3+^.

**Figure 9.**
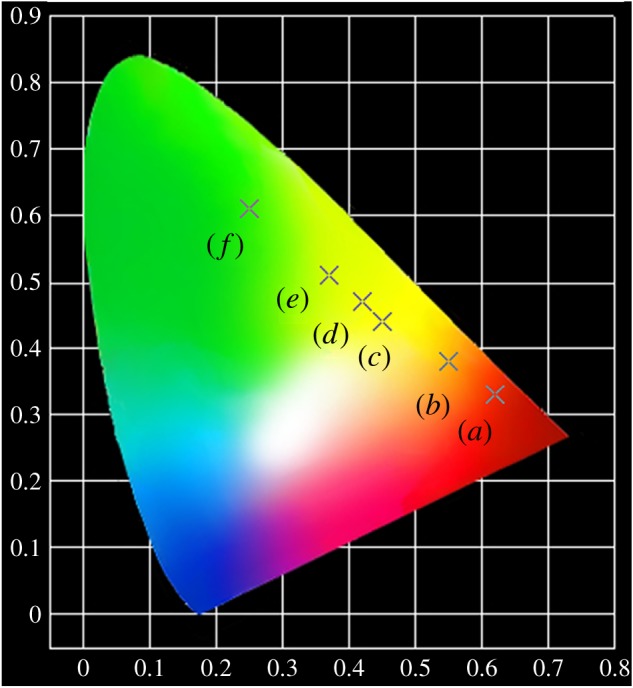
CIE chromaticity diagram for the emission spectra of the as-obtained Eu^3+^ and Tb^3+^ co-doped YBO_3_ samples: (*a*) YBO_3_: 5 mol% Eu^3+^; (*b*) YBO_3_: 4 mol% Eu^3+^, 1 mol% Tb^3+^; (*c*) YBO_3_: 3 mol% Eu^3+^, 2 mol% Tb^3+^; (*d*) YBO_3_: 2 mol% Eu^3+^, 3 mol% Tb^3+^; (*e*) YBO_3_: 1 mol% Eu^3+^, 4 mol% Tb^3+^; (*f*) YBO_3_: 5 mol% Tb^3+^.

In order to further illustrate the tunable PL property of the YBO_3_ sample, we co-doped Eu^3+^ and Tb^3+^ ions with different relative concentrations into the YBO_3_ host lattice (total concentration: 5 mol%). The emission spectra of the YBO_3_: *x* mol% Eu^3+^, (5 − *x*) mol% Tb^3+^ excited at 237 nm are depicted in figure 8 to show the succession of changes. It can be seen that the as-obtained YBO_3_: 5 mol% Eu^3+^ sample shows the characteristic emission peaks of Eu^3+^ ions. When Tb^3+^ ions were doped into the YBO_3_ host lattice, the YBO_3_: Eu^3+^/Tb^3+^ samples show not only the characteristic emission of Eu^3+^ ions, such as 590 nm (^5^D_0_ → ^7^F_1_), 610 and 624 nm (^5^D_0_ → ^7^F_1_), but also the characteristic emission of Tb^3+^ ions, such as 489 nm (^5^D_4_ → ^7^F_6_) and 541 nm (^5^D_4_ → ^7^F_5_). As one might expect, on increasing the relative concentration ratio of Eu^3+^/Tb^3+^, the luminescence of the Eu^3+^ ions gradually decreased, while that of Tb^3+^ increased. Finally, the pure YBO_3_: 5 mol% Tb^3+^ sample shows a bright green emission. As a result, the PL colour can be tuned from red, through orange, yellow and green-yellow, to green by simply adjusting the relative doping concentrations of the Eu^3+^ and Tb^3+^ ions. The result can be confirmed by the corresponding CIE chromaticity diagram for the emission spectra of the Eu^3+^ and Tb^3+^ co-doped YBO_3_ samples (figure 9). This result indicates that the as-obtained phosphors have the merit of multicolour emissions in the visible region when excited by a single wavelength of light, which might find potential applications in fields such as display systems and optoelectronic devices.

## 4. Conclusion

To sum up, YBO_3_ with a well-dispersed hollow microsphere shape has been successfully synthesized via the combination of a facile homogeneous precipitation approach, an ion-exchange process and a calcination process. The morphology, crystal structure and luminescence property of the as-obtained hollow microspheres were characterized by XRD, SEM, TEM and PL. Furthermore, the PL colour of the YBO_3_: Eu^3+^, Tb^3+^ samples can be controlled from red to orange to yellow to green-yellow and then to green by adjusting the relative doping concentrations of the activator ions, which indicates that the as-obtained phosphors could have the merit of multicolour emissions in the visible region when excited at a single wavelength. The material has a very important potential application in many fields, such as light display systems and optoelectronic devices, owing to its multicolour emission.

## Data accessibility

This article does not contain any additional data.
